# Adipose‐derived stem cell exosomes alleviate TGF‐β1‐induced urethral stricture fibrosis by suppressing the TGF‐β/Smad pathway and downstream PDGFR‐β/RAS/ERK signaling

**DOI:** 10.1002/ccs3.70025

**Published:** 2025-06-12

**Authors:** Tao Liang, Chao Deng, Hang Guo, Zhenghao Dai, Yiwen Jiang, Yuting Lu, Weiguo Chen

**Affiliations:** ^1^ Department of Urology The First Affiliated Hospital of Soochow University Suzhou Jiangsu China; ^2^ Department of Urology Shanghai Sixth People's Hospital Affiliated to Shanghai Jiao Tong University School of Medicine Shanghai China; ^3^ School of Clinical Medicine Shanghai University of Medicine and Health Sciences Shanghai China

**Keywords:** adipose stem cell exosomes, PDGFR‐β/RAS/ERK axis, TGF‐β/Smad signaling, urethral fibrosis, urethral stricture

## Abstract

This study aimed to investigate the therapeutic effects and underlying mechanisms of adipose‐derived stem cell exosomes (ADSCs‐exo) in ameliorating fibrosis in a rat model. ADSCs were isolated and cultured from rat adipose tissue, and ADSCs‐exo were extracted via ultracentrifugation. Urethral fibrosis was induced by local injection of TGF‐β1 (10 μg), followed by ADSCs‐exo treatment. Urodynamic parameters were evaluated, and histological changes were evaluated using hematoxylin and eosin and Masson staining. Transcriptomic analysis and pathway enrichment were performed to identify signaling pathways regulated by ADSCs‐exo. In vitro, urinary fibroblasts were stimulated with TGF‐β1 and treated with ADSCs‐exo alone or in combination with PDGF‐BB (agonist) or imatinib (inhibitor). ADSCs‐exo treatment significantly improved urodynamic function, reduced collagen deposition, and suppressed fibrosis‐related protein expression in vivo. Transcriptomic analysis revealed platelet‐derived growth factor and TGF‐β pathways as major contributors to fibrosis. In vitro, ADSCs‐exo significantly reduced TGF‐β1‐induced fibroblast proliferation, migration, and fibrosis‐related protein expression, effects that were reversed by PDGF‐BB and enhanced by imatinib. These findings were consistent in vivo, further supporting the hierarchical regulation of fibrosis‐related signaling by ADSCs‐exo. ADSCs‐exo mitigates urethral stricture fibrosis by primarily suppressing the TGF‐β/Smad pathway, thereby downregulating the downstream PDGFR‐β/RAS/ERK axis, highlighting its therapeutic potential as a cell‐free therapeutic approach for fibrotic urethral disease.

## Graphical abstract

Adipose‐derived stem cell exosomes alleviate TGF‐β1‐induced urethral fibrosis by inhibiting the PDGFR‐β/RAS/ERK signaling axis. This study reveals a dual regulatory mechanism through both the TGF‐β/Smad and platelet‐derived growth factor pathways, offering a promising noninvasive strategy for fibrotic disease treatment and urethral stricture prevention.

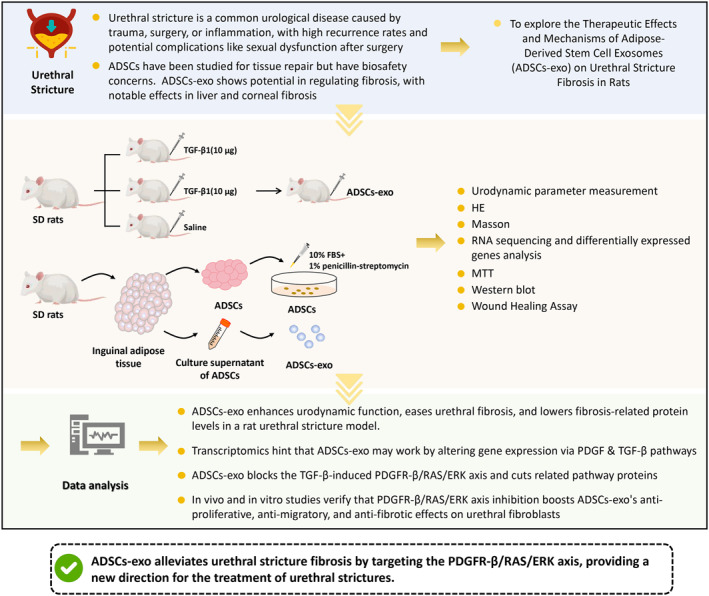

## 1 INTRODUCTION

Urethral stricture (US) represents a prevalent clinical condition within the field of urology. The high recurrence rate associated with this condition necessitates multiple surgical interventions, which impose significant physical, psychological, and financial burdens on patients.^1^ USs are predominantly observed in males, and the etiological factors contributing to this condition are multifaceted. Broadly, the causes can be categorized into four groups: inflammatory, traumatic, iatrogenic, and idiopathic, each exhibiting varying incidence rates.^2^ The frequency of traumatic and iatrogenic USs has been on the rise, correlating with an increase in traffic accidents and the expanding practice of endourological procedures. Notably, improper techniques during transurethral operations, catheterization, and other procedural interventions can lead to urethral injuries, thereby significantly elevating the incidence of USs.^3^ Research indicates that the onset and progression of USs not only result in lower urinary tract dysfunction but can also exacerbate this dysfunction, potentially leading to upper urinary tract complications and serious sequelae such as renal insufficiency, ultimately diminishing patients' quality of life.^4^

In clinical practice, the management of USs necessitates a comprehensive evaluation of various factors, including the patient's age, the underlying cause, the location and length of the stricture, the degree of fibrosis in the urethral and penile corpora cavernosa, any history of prior treatments, the preservation of sexual function, the presence of local infections, and the patient's overall nutritional status.^5^ Surgical intervention remains the primary treatment modality, encompassing procedures such as urethral incision and urethroplasty. For shorter strictures, such as those affecting the membranous urethra, endoscopic incision may be employed; however, this approach carries a risk of significant complications, including erectile and ejaculatory dysfunction, which can lead to treatment failure. Furthermore, the long‐term recurrence rate of strictures postsurgery remains notably high, with some urethral centers reporting rates as high as 26.2%.^6^, ^7^ This persistent challenge has prompted both domestic and international scholars to investigate strategies aimed at mitigating the complications associated with USs and to enhance treatment methodologies, yet effective reduction of recurrence rates has yet to be achieved.

The ongoing advancements in tissue engineering and stem cell technology have increasingly highlighted the significance of stem cells in the context of urethral repair. Mesenchymal stem cells are considered one of the most promising cellular sources for this purpose due to their robust differentiation capabilities, immunomodulatory functions, and their capacity to enhance local cell proliferation and differentiation. These stem cells can be cultured and expanded in vitro, allowing them to differentiate into various cell types within the urinary tract system, thus facilitating the remodeling of both the urethral mucosa and muscular layer.^8^ Among these, adipose‐derived stem cells (ADSCs), which are sourced from adipose tissue, present several advantages, including their abundant availability in the body, ease of access, and straightforward extraction and expansion processes, making them a focal point of research interest.^9^

Nonetheless, there are notable biosafety concerns associated with stem cell therapies, such as the potential instability of stem cells within the body and the associated risk of tumorigenesis.^10^ Recent studies have indicated that ADSCs may exert therapeutic effects in a range of diseases through the action of exosomes. Exosomes are nanoscale vesicles, measuring 30–150 nm, that are secreted by various living animal cells and are distributed across multiple systems and organs within the human body. Previous studies have shown that ADSC‐derived exosomes (ADSCs‐exo) can play a therapeutic role in a variety of diseases. ADSCs‐exo can regulate the regeneration and repair process of damaged parts by affecting biological functions such as cell proliferation, migration, and differentiation.^11^, ^12^ Additionally, research has demonstrated that ADSCs‐exo play a significant role in the treatment of several fibrotic conditions.^13^, ^14^ For instance, Wu et al. reported that ADSCs‐exo can effectively mitigate liver fibrosis by inhibiting the activation of hepatic stellate cells and modulating the metabolism of glutamine and ammonia mediated by hepatocyte glutamine synthetase.^15^ Similarly, Shen et al. found that ADSCs‐exo can downregulate HIPK2 expression by upregulating the delivery of miR‐19a, thereby slowing the differentiation of keratocytes into myofibroblasts in rabbit corneas.^16^ These findings underscore the potential of ADSCs‐exo as a signaling conduit in the treatment of fibrotic diseases. However, the specific role of ADSCs‐exo in the treatment of US, along with the underlying mechanisms of action, remains to be elucidated. Therefore, this study aims to explore the therapeutic potential of ADSCs‐exo in a rat model of TGF‐β1‐induced US fibrosis. By targeting the PDGFR‐β/RAS/ERK axis, the research seeks to elucidate key mechanisms underlying fibrosis reduction. Clinically, this approach could offer a minimally invasive alternative, lowering recurrence rates and improving patient outcomes while providing insights into broader applications of ADSCs‐exo in fibrotic disease management.

## 2 MATERIALS AND METHODS

### 2.1 Isolation and culture of ADSC cells

ADSCs were isolated from the inguinal fat of male SD rats. Three SD rats were purchased from Shanghai Rat&Mouse Biotech Co., Ltd and killed by intraperitoneal injection of 1.5% sodium pentobarbital solution and then fully disinfected with 75% alcohol. Then they were transferred to the clean bench, the skin on both sides of the abdomen was cut open, and the skin and subcutaneous tissue in the inguinal area were extensively separated to expose the inguinal fat pads on both sides. The corresponding fat pads were removed along the groin with the help of scissors. About 4 mL of tissue was removed from each rat. Then it was repeatedly rinsed with sterile PBS solution, and the fascia and blood vessels were removed with forceps and scissors. The adipose tissue was transferred to a sterile 5‐mL centrifuge tube and chopped into a paste. The same volume of 0.25% Type I collagenase solution was added for full digestion. After filtering through a 70‐μm cell strainer, the sample was centrifuged at 1000 *g* for 5 min to obtain a precipitate. Dulbecco's Modified Eagle Medium (DMEM) culture medium containing 10% fetal bovine serum and 1% penicillin‐streptomycin was added to resuspend the cells and then placed in a cell culture flask. They were transferred to a cell culture incubator, and adherent cells were cultured at 37°C, 5% CO_2_, and a certain humidity. The culture medium were replaced every 2 days.

### 2.2 Isolation of ADSCs‐exo

The culture supernatant of ADSCs was collected under sterile conditions and centrifuged at 4000 *g* for 30 min. The supernatant was removed and filtered through a 0.2‐μm polyethersulfone filter membrane. The filtrate was filtered through a tangential flow filtration system (Hanbon), then ultrafiltered through a 500 kDa mPES module, and finally filtered through a 0.1‐μm track‐etched membrane. The filtered sample was placed in a 100‐kDa ultrafiltration centrifuge tube and centrifuged at 4000 *g* for 10 min. The supernatant was removed to obtain a fully concentrated ADSCs‐exo sample.

### 2.3 Establishment of rat US fibrosis model

Eighteen SD rats were selected to establish a rat US fibrosis model. The rats were anesthetized by injection of 30 mg/kg sodium pentobarbital, fixed in a supine position, and the ventral penis skin was fully unfolded to observe the urethra. An epidural catheter was used as a rat urethral support tube and inserted into the urethra. Ten micrograms of TGF‐β1 was injected into the urethral wall at the upper, lower, left, and right positions with a 30‐gauge needle. Rats injected with the same amount of saline at the same position were used as the control group. Rats in the US group were injected with ADSCs‐exo at the same position of the urethral wall as the ADSCs‐exo group. After 4 weeks, the urodynamic parameters were measured, and the urethral tissue of the rats was obtained. The animal experiments described in this study were authorized by the Committee of the Experimental Animal Ethics Committee of Guangdong Medical Laboratory Animal Centre (D202412‐13).

### 2.4 Urodynamic parameter measurement

The treated rats were anesthetized by injecting 30 mg/kg of sodium pentobarbital. A small incision was made in the midline of the abdomen, and the abdominal wall tissue was gently separated to expose the bladder. A PE‐50 urinary catheter was connected to a pressure sensor and inserted into the bladder through the small incision at the top of the bladder. The urinary catheter was fixed to the bladder wall with sutures, and the other end of the catheter was connected to the pressure sensor and infusion pump of the urodynamic measurement system (Bonito XL). Physiological saline was infused into the bladder at a constant flow rate (e.g., 0.05–0.1 mL/min) through the infusion pump. When the rat bladder was infused to a certain volume, a urination reflex was triggered. The increase in intrabladder pressure was observed, and the peak bladder pressure during urination was recorded. The urine was discharged into the measuring container through the exposed end of the catheter to measure the urine volume. The volume of urinated liquid was measured with a pipette. The changes in bladder pressure over time were recorded in real time by the urodynamic measurement system, including the basal pressure during bladder filling, the peak pressure during urination, and the residual pressure after urination.

### 2.5 Hematoxylin and eosin (H&E) staining

Urethral tissue blocks, including the stenotic urethra, were cut and fixed in 4% paraformaldehyde for 48 h. The fixed tissue was rinsed with running water to remove impurities on the surface. Dehydration was performed in alcohol levels (50%, 70%, 85%, and 95% to anhydrous ethanol; each level took 2 h) to remove moisture from the tissue and then immersed in 100% xylene for permeabilization. The tissue was immersed in paraffin until it was completely solidified. The wax block was cut into slices with a thickness of 4 μm using a rotary slicer. The obtained slices were immersed in 100% xylene for 10 min for dewaxing and hydrated in alcohol levels (100%, 95%, 85%, 75%, and 50% alcohol were immersed in sequence for 5 min). The slides were stained with hematoxylin stain for 3 min, rinsed with running water, differentiated with 1% hydrochloric acid alcohol, stained with eosin for 2 min, and then the obtained slices were dehydrated with 70% alcohol. The above slides were placed in xylene for transparency for 3 min, repeated once, and then sealed with neutral gum. The cells were observed and photographed under an inverted microscope (CKX41, Nikon).

### 2.6 Masson staining

The above‐obtained sections were stained with Mayer hematoxylin staining solution for 3 min and rinsed with distilled water twice, and the obtained tissue was differentiated with 1% hydrochloric acid ethanol differentiation solution until it turned red and then rinsed with running water for 10 min. Staining was continued with fuchsin staining solution for 10 min, rinsed with distilled water twice, and phosphomolybdic acid solution was applied for 10 min. Aniline blue staining solution was added for 5 min. The tissue was dehydrated with 95% ethanol for 30 s, rendered transparent with xylene for 2 min, sealed with neutral gum, and examined under a microscope (CKX41, Nikon).

### 2.7 RNA sequencing and identification of DEGs

Total RNA was extracted from US tissues using TRIzol reagent following the manufacturer's protocol. Briefly, 100 mg of frozen tissue, ground into powder under liquid nitrogen, was lysed in 1 mL of TRIzol. After adding 200 μL of chloroform and centrifuging at 12,000*g* for 15 min at 4°C, the aqueous phase was collected. RNA was precipitated with isopropanol, washed with 75% ethanol, air‐dried, and dissolved in DEPC‐treated water. RNA purity and concentration were assessed using a NanoDrop spectrophotometer, and integrity was confirmed by agarose gel electrophoresis.

For RNA sequencing, cDNA libraries were prepared using the NEBNext Ultra RNA Library Prep Kit. Sequencing was performed on an Illumina platform, generating paired‐end reads. Clean reads were obtained by removing low‐quality reads, adapters, and contaminants using Trimmomatic software.

Differentially expressed genes (DEGs) were identified through comparative analysis using DESeq2, with thresholds of |log2 fold change| > 1 and adjusted *p* < 0.05. Kyoto Encyclopedia of Genes and Genomes (KEGG) pathway enrichment analyses were conducted using the clusterProfiler package in R.

### 2.8 Isolation and culture of primary UFs

Rats were anesthetized and euthanized by injection of 30 mg/kg sodium pentobarbital. The ventral penis skin was exposed, and the submucosal tissue of the urethra was removed to remove blood and excess tissue. The tissue was cut into small pieces (about 1–2 mm^3^) with sterile surgical scissors, and the tissue pieces were placed in a PBS solution containing 0.1% collagenase I and digested at 37°C for 1–2 h. A 0.25% trypsin solution was then added to continue digestion for 15–30 min, and the digested tissue suspension was filtered through a 70‐μm cell filter to collect the single cell suspension in the filtrate. The suspension was centrifuged at 1000*g* for 5 min, the cell pellet was resuspended in DMEM medium containing 10% fetal bovine serum, the cell suspension was inoculated into a sterile culture bottle precoated with culture medium. The cells were cultured in an incubator at 37°C and 5% CO_2_, and then the medium was changed every 2–3 days. Urinary fibroblasts were seeded in 96‐well or 6‐well plates at an appropriate density and allowed to adhere to the wall and reach 70%–80% confluence. Urethral fibroblasts (UFs) were treated with normal culture medium, TGF‐β1 (10 ng/mL), TGF‐β1 (10 ng/mL) + ADSCs‐exo, TGF‐β1 (10 ng/mL) + ADSCs‐exo + PDGF‐BB (100 ng/mL), and TGF‐β1 (10 ng/mL) + ADSCs‐exo + imatinib (10 μM) for 24 h for subsequent experiments.

### 2.9 MTT assay

Urinary fibroblasts were evenly seeded in a 96‐well plate at 5 × 10^4^ cells/well. Intervention was performed according to the experimental design. After the treatment, 10 μL of 3‐(4,5‐dimethylthiazol‐2‐yl)‐2,5‐diphenyl tetrazolium bromide (MTT) solution (C0009S, Beyotime) was added to each well to reach a final concentration of 0.5 mg/mL. The 96‐well plate was returned to the incubator and incubated for 3–4 h. The light absorption value (OD value) of each well was measured at a wavelength of 570 nm using a Synergy2 microplate reader (BioTek).

### 2.10 Scratch assay

Urinary fibroblasts were evenly seeded in 6‐well plates at 1 × 10^5^ cells/well and formed a monolayer with approximately 80%–90% confluence within 24 h. A sterile 200‐μL pipette tip was used to draw a uniform straight line in the cell monolayer perpendicular to the surface of the culture plate. The cells were slowly rinsed twice with sterile PBS. Different treatments were added according to the experimental design, and the 6‐well plates were returned to the incubator for further incubation. Cell migration and wound healing were observed for 24 h in the different treatment groups. Image processing software such as ImageJ was used to quantify the changes in the scratch area.

### 2.11 Western blot

Total protein was extracted from cells or urethral tissues using radioimmunoprecipitation assay buffer lysis buffer mixed with phenylmethylsulfonyl fluoride. The protein concentration was determined using a BCA kit (P0009, Beyotime). Twenty micrograms of protein were separated using 10% SDS‐PAGE and then transferred to a polyvinylidene fluoride (PVDF) membrane by wet transfer at 90 V for 1 h. The PVDF membrane was immersed in 5% skim milk powder tris‐buffered saline with Tween 20 (TBST) solution. Then, according to the instructions, the antibodies anti‐Col I (1:1000, ab316222, Abcam), anti‐Col III (1:1000, ab184993, Abcam), anti‐α‐SMA (1:1000, ab7817, Abcam), antifibronectin (1:1000, ab2413, Abcam), anti‐TGF‐β1 (1:1000, ab215715, Abcam), anti‐Smad2 (1:1000, ab40855, Abcam), anti‐p‐Smad2 (1:1000, ab280888, Abcam), anti‐Smad3 (1:1000, ab40854, Abcam), anti‐p‐Smad3 (1:1000, ab52903, Abcam), anti‐PDGFA (1:1000, ab203911, Abcam), anti‐PDGFB (1:1000, ab178409, Abcam), anti‐PDGFR‐α (1:1000, ab203419, Abcam), anti‐p‐PDGFR‐α (1:1000, ab134068, Abcam), anti‐PDGFR‐β (1:1000, ab313777, Abcam), anti‐p‐PDGFR‐β (1:1000, ab218534, Abcam), anti‐MEK1/2 (1:1000, ab178876, Abcam), anti‐p‐MEK1/2 (1:1000, ab278564, Abcam), anti‐ERK1/2 (1:1000, ab184699, Abcam), anti‐p‐ERK1/2 (1:1000, ab126455, Abcam), and glyceraldehyde‐3‐phosphate dehydrogenase (GAPDH) (1:1000, ab8245, Abcam) were diluted in proportion and incubated at 4°C overnight. After the PVDF membrane incubated with the primary antibody was washed three times with TBST, it was incubated with the secondary antibody (goat anti‐rabbit/mouse IgG H&L/HRP antibody [1:10,000, ab6721/ab205719, Abcam]) diluted in TBST solution for 1 h. The PVDF membrane was immersed in enhanced chemiluminescence luminescent solution (P0018S, Beyotime) for 1 min, and the image was taken using an imaging system (Alpha Innotech). The relative expression level of the target protein was analyzed using ImageJ software using GAPDH.

### 2.12 Statistical methods

The experimental results were expressed as mean ± standard deviation, and the graphs and significance analysis were performed using GraphPad Prism 9.0 software. The independent‐sample *t*‐test was used for comparisons between two groups, and one‐way analysis of variance was used for comparisons among multiple groups. *p* < 0.05 was considered statistically significant.

## 3 RESULT

### 3.1 ADSCs‐exo alleviate TGF‐β1‐induced US fibrosis in rats

**FIGURE 1:**
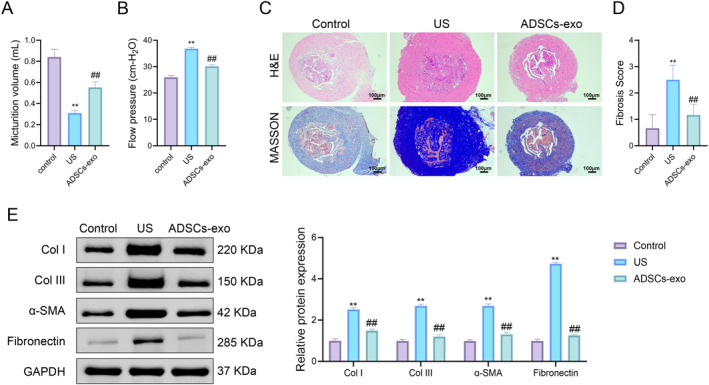
ADSCs‐exo alleviates TGF‐β1‐induced US fibrosis in rats. (A) Evaluation of urine output in the control, US, and ADSCs‐exo groups. (B) Evaluation of flow pressure in the control, US, and ADSCs‐exo groups. (C) H&E staining and Masson staining were used to observe the histological changes and the fibrosis level of the urethral tissues. (D) Fibrosis scores were performed on the urethral tissues. (E) Western blot was used to detect the protein expression levels of Col I, Col III, α‐SMA, and fibronectin in the urethral tissues. Data are presented as mean ± SD. **p < 0.01 versus control; ^##^ *p* < 0.01 versus US. ADSCs‐exo, adipose‐derived stem cell exosomes; H&E, hematoxylin and eosin; US, urethral stricture.

To observe the effect of ADSCs‐exo on rat US fibrosis, a rat US fibrosis model was established by injecting TGF‐β1. Compared with the control group, the urine output of rats in the US group was significantly reduced, and the flow pressure was significantly increased; after ADSCs‐exo treatment, the urine output was significantly increased, and the flow pressure was significantly reduced (Figure 1A,B). According to H&E staining, the urethral tissue structure of rats in the control group was normal, and there was no submucosal fibrosis, whereas rats in the US group had dense collagen bundles, sparse smooth muscle, and moderate fibrosis. After ADSCs‐exo treatment, the tissue structure of the urethral mucosal injury site of US rats was generally normal, the epithelial layer was well formed, and only mild fibrosis was present (Figure 1C). The collagen volume of the tissue was evaluated by Masson staining, and the results showed that the collagen volume of the US group was significantly higher than that of the control group, whereas the collagen volume of the ADSCs‐exo group was significantly lower than that of the US group (Figure 1C,D). According to Western blot, TGF‐β1 can increase the expression of fibrosis‐related proteins in rat urethral tissue, whereas ADSCs‐exo can reduce the protein expression levels of Col I, Col III, α‐SMA, and fibronectin (Figure 1E). The results showed that TGF‐β1 injection successfully induced urethral fibrosis in rats, and ADSCs‐exo treatment could significantly improve urodynamics and reduce urethral fibrosis in rats.

### 3.2 Transcriptomic profiling reveals dual regulation of the TGF‐β/Smad and platelet‐derived growth factor (PDGF) pathways by ADSCs‐exo in urethral fibrosis

**FIGURE 2:**
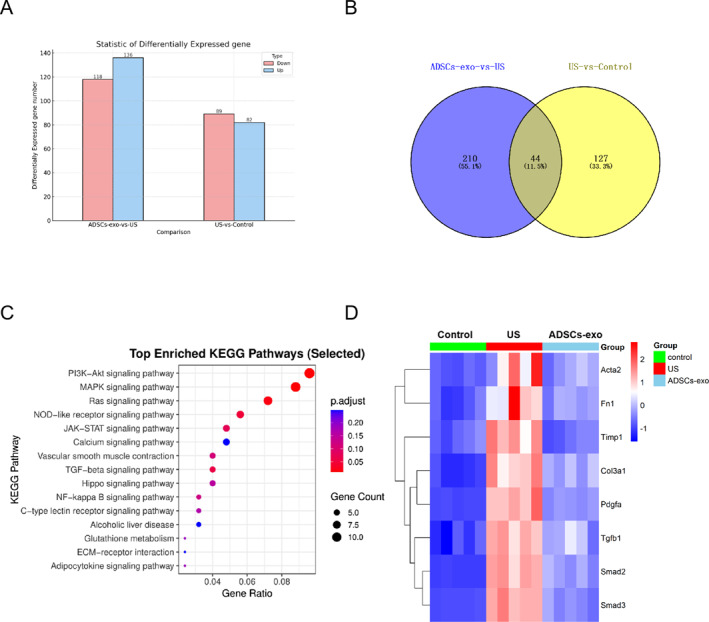
Transcriptomic analysis of TGF‐β1‐induced US fibrosis in rats and ADSCs‐exo treatment. (A) DEGs were identified between the control group and the US group and between the US group and the ADSCs‐exo group. (B) A Venn diagram demonstrated DEGs shared among the three groups. (C) KEGG enrichment analysis of these shared DEGs. (D) Heatmap of the top 8 shared DEGs. Each column represents an individual sample from the control (green), US (red), or ADSCs‐exo (blue) groups. ADSCs‐exo, adipose‐derived stem cell exosomes; DEGs, differentially expressed genes; KEGG, Kyoto Encyclopedia of Genes and Genomes; US, urethral stricture.

To explore the molecular mechanisms underlying the antifibrotic effects of ADSCs‐exo, transcriptome sequencing technology (RNA‐seq) was performed on urethral tissues from the control, US, and ADSCs‐exo groups. A total of 171 DEGs (82 upregulated, 89 downregulated) in the US versus control comparison and 254 DEGs (136 upregulated, 118 downregulated) in the ADSCs‐exo versus US comparison were identified (Figure 2A). A total of 44 overlapping DEGs were identified between the two comparisons, representing key fibrosis‐related genes modulated by both US and ADSCs‐exo intervention (Figure 2B, Table S1). KEGG pathway enrichment analysis (Figure 2C) of these shared DEGs revealed that the most significantly enriched signaling pathways were the PI3K‐Akt, MAPK, and Ras pathways—all of which are directly regulated by PDGF—as well as the TGF‐β signaling pathway, which plays a pivotal upstream role in fibrogenesis.^17^, ^18^ Heatmap analysis confirmed that these fibrosis‐related genes were highly upregulated in the US group and were notably downregulated after ADSCs‐exo treatment (Figure 2D).

These findings suggested that ADSCs‐exo might exert its therapeutic effects by coordinately modulating both PDGF and TGF‐β/Smad signaling pathways.

### 3.3 Effects of ADSCs‐exo on TGF‐β/Smad and downstream PDGF‐β/RAS/ERK signaling in TGF‐β1‐induced urethral fibrosis

TGF‐β1 is a key profibrotic cytokine that promotes tissue fibrosis primarily through activation of the canonical TGF‐β/Smad pathway and also enhances PDGF expression. PDGF signaling, in turn, activates downstream pathways such as the RAS/ERK cascade, which regulates fibroblast proliferation, migration, and extracellular matrix remodeling.^19^ Given this signaling hierarchy, we assessed the activation status of the TGF‐β/Smad, PDGF, and RAS/ERK signaling pathways in urethral tissues.

**FIGURE 3:**
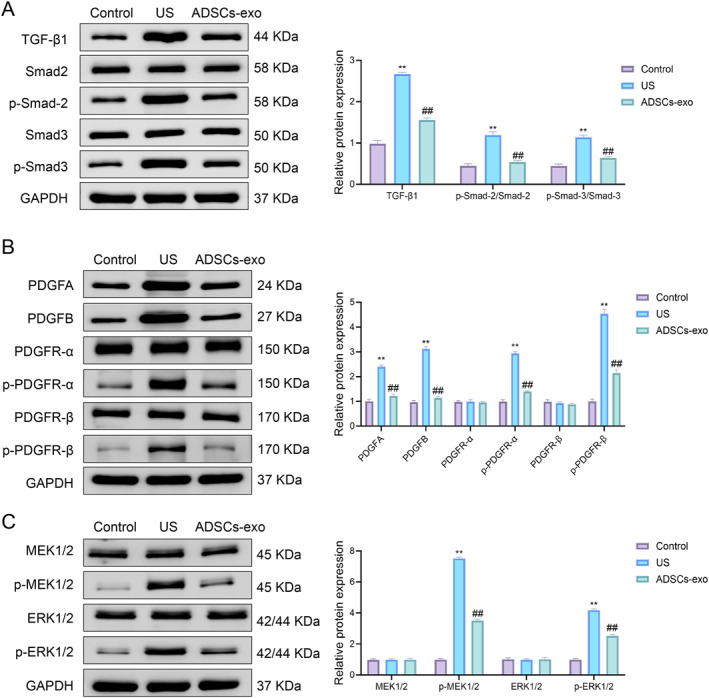
Effects of ADSCs‐exo treatment on TGF‐β/Smad pathway, PDGF signaling pathway, and downstream RAS/ERK pathway in rats. (A) Western blot was used to detect the protein levels of TGF‐β1, Smad2, p‐Smad2, Smad3, and p‐Smad3 in the urethral tissues of rats in the control group, US group, and ADSCs‐exo group. (B) Western blot was used to detect the protein levels of PDGFA, PDGFB, PDGFR‐α, p‐PDGFR‐α, PDGFR‐β, and p‐PDGFR‐β in the urethral tissues. (C) Western blot was used to detect the protein expression levels of MEK1/2, p‐MEK1/2, ERK1/2, and p‐ERK1/2 in the urethral tissues. Data are presented as mean ± SD. **p < 0.01 versus control; ^##^ *p* < 0.01 versus US. ADSCs‐exo, adipose‐derived stem cell exosomes; PDGF, platelet‐derived growth factor; US, urethral stricture.

The expression of TGF‐β1, p‐Smad2/Smad2, and p‐Smad3/Smad3 was elevated in the US group compared to the control, whereas ADSCs‐exo treatment notably reduced these levels (Figure 3A). Similarly, PDGF pathway components—including PDGFA, PDGFB, p‐PDGFR‐α, and p‐PDGFR‐β—were upregulated in the US group and were significantly suppressed by ADSCs‐exo (Figure 3B).

To further investigate downstream signaling, we measured RAS/ERK pathway activation by assessing the phosphorylation levels of MEK1/2 and ERK1/2. These were markedly increased in the US group, indicating pathway activation. ADSCs‐exo treatment significantly reduced the phosphorylation levels of MEK1/2 and ERK1/2 proteins (Figure 3C).

Together, these findings indicated that the therapeutic effect of ADSCs‐exo was associated with the suppression of multiple fibrosis‐related pathways, including the TGF‐β/Smad and PDGFR‐β/RAS/ERK axes.

### 3.4 ADSCs‐exo suppresses proliferation, migration, and fibrosis of TGF‐β1‐induced UFs via the PDGFR‐β/RAS/ERK axis

To further investigate the role of the PDGFR‐β/RAS/ERK signaling axis in ADSCs‐exo‐mediated antifibrotic effects, TGF‐β1‐induced UFs were treated with PDGFR agonist PDGF‐BB or inhibitor imatinib.

**FIGURE 4:**
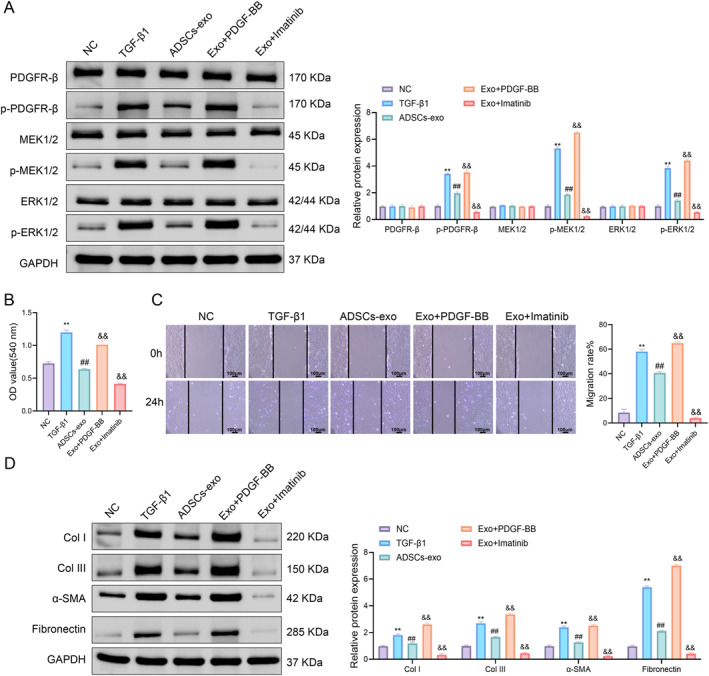
Inhibition of the PDGFR‐β/RAS/ERK axis enhances the antiproliferative, antimigratory, and antifibrotic effects of ADSCs‐exo on TGF‐β1‐induced urethral fibroblasts. (A) Western blot was used to detect the protein expression levels of PDGFR‐β, p‐PDGFR‐β, MEK1/2, p‐MEK1/2, ERK1/2, and p‐ERK1/2. (B) MTT was used to detect the viability of rat urethral fibroblasts. (C) Scratch assay showed the migration ability of rat urethral fibroblasts. (D) Western blot analysis of fibrotic markers (Col I, Col III, α‐SMA, and fibronectin). Data are presented as mean ± SD. **p < 0.01 versus NC; ^##^ *p* < 0.01 versus TGF‐β1; ^&&^ *p* < 0.01 versus ADSCs‐exo. ADSCs‐exo, adipose‐derived stem cell exosomes; MTT, 3‐(4,5‐dimethylthiazol‐2‐yl)‐2,5‐diphenyl tetrazolium bromide; NC, negative control.

Western blot analysis showed that compared to ADSCs‐exo alone, PDGF‐BB restored the phosphorylation of PDGFR‐β, MEK1/2, and ERK1/2, whereas imatinib further suppressed their activation (Figure 4A). Functional assays revealed that TGF‐β1 significantly enhanced UF viability and migration, which were attenuated by ADSCs‐exo. These effects were partially reversed by PDGF‐BB and further enhanced by imatinib (Figure 4B,C). Furthermore, ADSCs‐exo markedly reduced the expression of fibrotic markers, including Col I, Col III, α‐SMA, and fibronectin, whereas PDGF‐BB increased and imatinib decreased their levels compared to the ADSCs‐exo group (Figure 4D).

These results indicated that inhibiting the PDGFR‐β/RAS/ERK axis potentiated the antiproliferative, antimigratory, and antifibrotic effects of ADSCs‐exo in TGF‐β1‐induced UFs.

### 3.5 Inhibition of the PDGFR‐β/RAS/ERK axis enhances the antifibrotic effect of ADSCs‐exo on TGF‐β1‐induced rats in vivo

**FIGURE 5:**
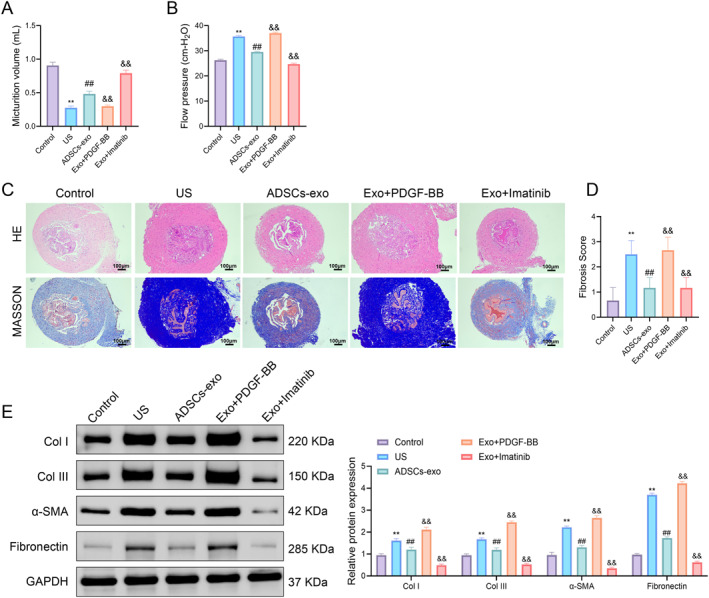
Inhibition of the PDGFR‐β/RAS/ERK axis enhances the antifibrotic effect of ADSCs‐exo on TGF‐β1‐induced rats in vivo. (A) Evaluation of urine output of rats in the control group, US group, ADSCs‐exo group, exo + PDGF‐BB group, and exo + imatinib group. (B) Evaluation of flow pressure of rats in each group. (C) H&E staining and Masson staining were used to observe the histological changes and fibrosis level of urethral tissues of rats. (D) Fibrosis scores were performed on urethral tissues of rats. (E) Western blot was used to detect the protein expression levels of Col I, Col III, α‐SMA, and fibronectin in the urethral tissues of rats. Data are presented as mean ± SD. **p < 0.01 versus control; ^##^ *p* < 0.01 versus US; ^&&^ *p* < 0.01 versus ADSCs‐exo. ADSCs‐exo, adipose‐derived stem cell exosomes; H&E, hematoxylin and eosin; US, urethral stricture.

To validate the in vitro findings, we conducted in vivo experiments in TGF‐β1‐induced US rats treated with ADSCs‐exo in combination with PDGF‐BB or imatinib. Urodynamic analysis revealed that PDGF‐BB attenuated the therapeutic effects of ADSCs‐exo, leading to reduced urine volume and increased flow pressure. In contrast, imatinib enhanced these parameters relative to the ADSCs‐exo group (Figure 5A,B). Histological evaluation showed that urethral fibrosis was exacerbated in the exo + PDGF‐BB group, with denser collagen bundles and narrowed lumens, whereas fibrosis was further attenuated in the exo + imatinib group, as confirmed by H&E and Masson staining and corresponding fibrosis scores (Figure 5C,D). Western blot analysis showed that PDGF‐BB upregulated fibrotic markers (Col I, Col III, α‐SMA, and fibronectin), whereas imatinib further downregulated these proteins compared to ADSCs‐exo alone (Figure 5E).

These findings confirmed that blocking the PDGFR‐β/RAS/ERK axis enhanced the antifibrotic efficacy of ADSCs‐exo in vivo.

## 4 DISCUSSION

ADSCs‐exo are subcellular entities found within the lipid bilayer membrane of ADSCs. These exosomes are enriched with a diverse array of bioactive compounds that mirror those present in the parent cells, thereby facilitating the repair of damaged cells. The findings of this study indicate that ADSCs‐exo possess advantages in terms of size and membrane structure, demonstrating superior safety profiles compared to traditional adipose stem cell therapies in various animal model studies addressing conditions affecting the kidneys, heart, nervous system, and skin. Furthermore, the therapeutic efficacy of ADSCs‐exo surpasses that of transplanted adipose stem cells.^20^, ^21^ Consequently, this research seeks to investigate the application of ADSCs‐exo therapy in the context of urethral repair, thereby exploring a novel approach to this clinical challenge.

The process of fibrosis associated with US is primarily driven by TGF‐β1, which has been identified as a significant contributor to various fibrotic diseases.^22^ TGF‐β1 facilitates the development of fibrotic lesions by stimulating fibroblasts to produce extracellular matrix proteins, including collagen and fibronectin. Additionally, it enhances the expression of extracellular matrix degradation inhibitors, such as TIMP, thereby increasing the stability of fibrotic lesions.^23^ Consequently, this study employed TGF‐β1 as an inducer to establish a rat model of US fibrosis. Exosomes serve as mediators of intercellular communication, capable of releasing vesicles that contain proteins and nucleic acids, and are thought to play a significant role in cellular fibrosis.^24^ These exosomes can convey microRNA signaling molecules that inhibit fibrosis and downregulate the expression of fibrosis‐related proteins in target cells, thereby influencing the fibrotic process.^25^ In our study, ADSCs‐exo can markedly ameliorate urethral mucosal injury and reduce fibrotic collagen deposition in rats with US while simultaneously decreasing the expression levels of fibrosis‐associated proteins.

Transcriptomic profiling revealed that urethral fibrosis in TGF‐β1‐treated rats involved multiple signaling pathways, including both TGF‐β/Smad and PDGF signaling, and that these were significantly downregulated following ADSCs‐exo treatment. This dual‐pathway modulation highlights the complex network of signaling events involved in fibrosis. Importantly, our findings suggest that ADSCs‐exo primarily targets the TGF‐β/Smad pathway, as evidenced by reduced expression of TGF‐β1 and decreased phosphorylation of Smad2 and Smad3. These Smad proteins are well‐known transcription factors that upregulate PDGFRs and PDGF ligands, thereby enhancing downstream PDGF responsiveness.^17^ Therefore, the observed inhibition of PDGFA, PDGFB, and phosphorylated PDGFR‐β following ADSCs‐exo treatment may be a secondary effect of upstream suppression of TGF‐β signaling.

The RAS/RAF/MEK/ERK signaling cascade, downstream of PDGFR activation, is a well‐characterized signaling cascade involved in cell growth, migration, and survival.^26^ In the context of fibrosis, this pathway enhances fibroblast proliferation, ECM production, and cytoskeletal remodeling.^27^, ^28^, ^29^ Our data demonstrated that the RAS/ERK pathway was significantly activated in the US model and subsequently attenuated by ADSCs‐exo treatment. However, in line with the hierarchical relationship between these pathways, we propose that ADSCs‐exo exerts its primary antifibrotic effects by targeting TGF‐β/Smad signaling, which in turn downregulates PDGF ligand and receptor expression, leading to reduced activity of the PDGFR‐β/RAS/ERK cascade.

Based on the phenomenon of in vivo experiments, this study took PDGF as the research object and selected the PDGF agonist PDGF‐BB and inhibitor imatinib.^30^, ^31^ In vitro studies found that PDGF‐BB significantly inhibited the function of ADSCs‐exo, whereas imatinib enhanced the effect of ADSCs‐exo. This phenomenon emphasizes that ADSCs‐exo functions through the PDGFR‐β/RAS/ERK axis. It is known that in the proteome sequencing of ADSCs‐exo, the content of decorin and BMP was detected.^32^ Decorin is an extracellular matrix protein that binds to PDGF and prevents it from binding to the PDGF receptor, thereby inhibiting the activation of the PDGF signaling pathway.^33^ Decorin can also bind to TGF‐β and play an antifibrotic role in fibrosis.^34^ BMP can antagonize PDGF‐induced cell proliferation and migration.^35^ These proteins may be one of the reasons for inhibiting the activity of the PDGFR‐β/RAS/ERK axis. In addition, a variety of noncoding RNAs carried by ADSCs‐exo are also factors that regulate PDGF activity, including miRNA let‐7d, circCDK14, and lncRNA LnRPT.^36^, ^37^, ^38^ These components likely act synergistically to inhibit both the upstream TGF‐β pathway and its downstream PDGF signaling axis.

Despite promising findings, several limitations must be acknowledged. First, the investigation did not assess the therapeutic effects of ADSCs‐exo on various US models. Additionally, the signaling pathways associated with US fibrosis are intricate and exhibit interconnectivity. This study focused solely on the signaling axis of a single component, necessitating further research to determine whether ADSCs‐exo engages additional signaling pathways. Furthermore, a more in‐depth exploration is required to elucidate the specific molecular mechanisms through which ADSCs‐exo modulates the activity of the PDGFR‐β/RAS/ERK signaling axis.

In summary, our findings support a revised mechanistic interpretation in which ADSCs‐exo primarily targets the TGF‐β/Smad pathway, thereby indirectly inhibiting PDGFR‐β/RAS/ERK and attenuating fibrotic progression. This integrated pathway suppression underscores the therapeutic potential of ADSCs‐exo in treating US fibrosis.

## 5 CONCLUSION

This study suggests that ADSCs‐exo exerts its antifibrotic effects primarily through the suppression of the TGF‐β/Smad signaling pathway, which in turn downregulates the expression and activity of the PDGFR‐β/RAS/ERK axis. This hierarchical signaling model emphasizes TGF‐β as the upstream regulator and identifies the PDGF pathway as a downstream amplifier of fibrosis. These insights provide a more comprehensive understanding of the signaling interconnectivity in US and establish TGF‐β as a primary therapeutic target of ADSCs‐exo.

## AUTHOR CONTRIBUTIONS

All authors contributed to the study conception and design. Material preparation, data collection and analysis were performed by Hang Guo, Zhenghao Dai, Yiwen Jiang and Yuting Lu. The first draft of this manuscript was written by Tao Liang, Chao Deng and Weiguo Chen and all authors commented on previous versions of the manuscript. All authors read and approved the final manuscript.

## CONFLICT OF INTEREST STATEMENT

The authors declare no conflicts of interest.

## CONSENT FOR PUBLICATION

Not applicable.

## ETHICS STATEMENT

The animal experiments described in this study were authorized by the Committee of the Experimental Animal Ethics Committee of Shanghai Rat&Mouse Biotech Co., Ltd (RM202405 (41)).

## Supporting information

Table S1

## Data Availability

The datasets used and/or analyzed during this study are available from the corresponding author upon reasonable request.
